# Risk of Recurrent Venous Thromboembolism in Patients with Cancer: An Individual Patient Data Meta-analysis and Development of a Prediction Model

**DOI:** 10.1055/a-2418-3960

**Published:** 2024-10-16

**Authors:** Vincent R. Lanting, Toshihiko Takada, Floris T. M. Bosch, Andrea Marshall, Michael A. Grosso, Annie M. Young, Agnes Y. Y. Lee, Marcello Di Nisio, Gary E. Raskob, Pieter W. Kamphuisen, Harry R. Büller, Nick van Es

**Affiliations:** 1 Amsterdam UMC, University of Amsterdam, Department of Vascular Medicine, Amsterdam Cardiovascular Sciences, Amsterdam, The Netherlands; 2 Department of Internal Medicine, Tergooi Hospital, Hilversum, The Netherlands; 3 Julius Center for Health Sciences and Primary Care, University Medical Center Utrecht, Utrecht University, Utrecht, The Netherlands; 4 Department of General Medicine, Shirakawa Satellite for Teaching and Research (STAR), Fukushima Medical University, Fukushima, Japan; 5 Warwick Clinical Trials Unit, University of Warwick, Coventry, United Kingdom; 6 Clinical Development, Daiichi Sankyo, Basking Ridge, New Jersey, United States; 7 Clinical Trials Unit, Warwick Medical School, University of Warwick, Coventry, United Kingdom; 8 Division of Hematology, University of British Columbia, British Columbia Cancer Agency, Vancouver BC, Canada; 9 Department of Medicine and Ageing Sciences, Gabriele D′Annunzio University, Chieti, Italy; 10 University of Oklahoma Health Sciences Center and OU Health, Oklahoma City, Oklahoma, United States

**Keywords:** recurrent VTE, cancer, prediction

## Abstract

**Background:**

About 7% of patients with cancer-associated venous thromboembolism (CAT) develop a recurrence during anticoagulant treatment. Identification of high-risk patients may help guide treatment decisions.

**Aim:**

To identify clinical predictors and develop a prediction model for on-treatment recurrent CAT.

**Methods:**

For this individual patient data meta-analysis, we used data from four randomized controlled trials evaluating low-molecular-weight heparin or direct oral anticoagulants (DOACs) for CAT (Hokusai VTE Cancer, SELECT-D, CLOT, and CATCH). The primary outcome was adjudicated on-treatment recurrent CAT during a 6-month follow-up. A clinical prediction model was developed using multivariable logistic regression analysis with backward selection. This model was validated using internal–external cross-validation. Performance was assessed by the c-statistic and a calibration plot.

**Results:**

After excluding patients using vitamin K antagonists, the combined dataset comprised 2,245 patients with cancer and acute CAT who were treated with edoxaban (23%), rivaroxaban (9%), dalteparin (47%), or tinzaparin (20%). Recurrent on-treatment CAT during the 6-month follow-up occurred in 150 (6.7%) patients. Predictors included in the final model were age (restricted cubic spline), breast cancer (odds ratio [OR]: 0.42; 95% confidence interval [CI]: 0.20–0.87), metastatic disease (OR: 1.44; 95% CI: 1.01–2.05), treatment with DOAC (OR: 0.66; 95% CI: 0.44–0.98), and deep vein thrombosis only as an index event (OR: 1.72; 95% CI: 1.31–2.27). The c-statistic of the model was 0.63 (95% CI: 0.54–0.72) after internal–external cross-validation. Calibration varied across studies.

**Conclusion:**

The prediction model for recurrent CAT included five clinical predictors and has only modest discrimination. Prediction of recurrent CAT at the initiation of anticoagulation remains challenging.

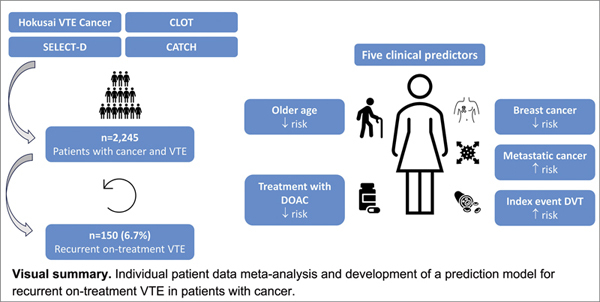

## Introduction

Venous thromboembolism (VTE), comprising deep vein thrombosis (DVT) and pulmonary embolism (PE), is a frequent complication in patients with cancer. 1 Direct oral anticoagulants (DOACs) or low-molecular-weight heparin (LMWH) are recommended for the treatment of acute VTE, 2 3 4 5 6 but the risk of recurrence nonetheless remains high. 7 In a meta-analysis of six randomized controlled trials (RCTs), the cumulative incidences of recurrent VTE over a 6-month treatment period were 5.4 and 8.3% in patients receiving DOAC or LMWH, respectively. 8

Patients with cancer and acute VTE are usually treated for at least 3 to 6 months. Anticoagulation is usually continued in case of active cancer or ongoing anticancer treatment. Decisions about the optimal intensity and duration of anticoagulant treatment should ideally be guided by the risk of recurrent VTE. For example, while in the RCTs the dose of LMWH was typically reduced by 25% after the first month of treatment to mitigate the risk of bleeding, but it is unknown if this dose reduction strategy should be avoided in cancer patients at high risk of recurrent VTE. Currently, the only risk stratification tool to determine the risk of recurrent VTE in cancer patients is the Ottawa score, which stratifies the risk of recurrence based on tumor type, cancer stage, and history of VTE. 9 However, several studies have shown poor discrimination of this score (c-statistics ranging from 0.5 to 0.7), which has limited its use in clinical practice. 10 11 In addition, this score provides a risk classification rather than an individualized risk estimate. Therefore, we sought to derivate and validate a novel clinical prediction model for recurrent VTE in cancer patients with acute VTE.

## Methods

### Study Selection

This report adheres to the Transparent Reporting of a multivariable prediction model for Individual Prognosis or Diagnosis (TRIPOD) guidance for individual patient data (IPD) meta-analysis ( Supplementary Table S1 , available in the online version). 12 We identified RCTs that evaluated anticoagulant treatment in patients with cancer and acute VTE up to 2021 based on previously published systematic reviews. 7 13 Studies were eligible if they included adult patients with active cancer (other than basal-cell or squamous-cell skin cancer) and acute symptomatic or incidental DVT or PE, and had at least 6 months of follow-up. Of eight identified trials 2 3 4 5 6 14 15 16 ( Supplementary Table S2 , available in the online version), IPD were obtained from four studies: Hokusai VTE cancer trial, 2 SELECT-D, 3 CATCH, 14 and CLOT. 15 These trials enrolled patients between 1999 and 2016. In all studies, active cancer was defined as a cancer diagnosis or cancer treatment in the 6 months prior to the first VTE event, or the presence of recurrent, regionally advanced, or metastatic solid cancer, or hematological cancer not in remission. The primary efficacy outcome was symptomatic or incidentally detected recurrent VTE in Hokusai VTE cancer and SELECT-D, while only symptomatic events were considered in the primary efficacy outcome of the CLOT and CATCH studies. In CLOT and CATCH, a vitamin K antagonist was compared with LMWH (dalteparin and tinzaparin, respectively), while Hokusai VTE cancer and SELECT-D trials compared an oral factor Xa inhibitor (edoxaban or rivaroxaban, respectively) with LMWH (dalteparin). Since vitamin K antagonists are no longer recommended as treatment for cancer-associated thrombosis, 17 18 19 patients allocated to these agents were excluded from the present analysis. The primary outcome was recurrent on-treatment VTE, which was defined a symptomatic or incidentally detected DVT or PE that was diagnosed during use of study treatment. In the original studies, all outcome events were adjudicated without knowledge of treatment allocation. In the present analysis, only events that were adjudicated by the original study as being on-treatment were included. The definition of the on-treatment period was from randomization until 24 to 72 hours after last intake of study drug.

### Selection of Candidate Predictors and Model Development

Candidate predictors were selected based on their known association with a first or recurrent VTE in the literature and their availability in the databases. 20 21 22 Based on the (modified) Ottawa score, breast and lung cancers were evaluated as binary predictors. In addition, we also evaluated cancer types associated with the risk of a first VTE, including hepatobiliary cancer, gynecological cancer, hematological cancer, and genitourinary cancer excluding prostate cancer. In an explorative analysis, cancer type was categorized based on the risk of a first VTE using the classification proposed by Li and colleagues that includes 23 very high-risk cancer (pancreatic, gastroesophageal, bile duct, and gall bladder cancer), high-risk cancer (lung, ovarian, uterine, bladder, kidney, testicular, primary brain cancer, aggressive non-Hodgkin lymphoma, multiple myeloma, and soft tissue sarcoma), intermediate-risk cancer (colorectal cancer), and low-risk cancer (all other cancers). Other candidate predictors included age (continuous), sex, body weight (continuous), platelet count of >350 × 10 ^9^ /L, use of antiplatelet agents, type of anticoagulant treatment (LMWH vs. DOAC), and index VTE type (PE with or without DVT vs. DVT only). The following candidate predictors were identified but could not be used because they were not available in all databases: hemoglobin level, leukocyte count, smoking, ethnicity, anticancer treatment, and plasma creatinine. Partially missing data for candidate predictors up to 15% were imputed within studies using multiple imputation with chained equations, using a model that included most baseline variables as well as outcomes. 24 Systematically missing data were not imputed.

Candidate predictors were first evaluated in a univariable logistic regression model within each study. Odds ratios (ORs) were pooled in a random-effects meta-analysis using the Hartung–Knapp method. Between-study heterogeneity was assessed for each predictor and displayed using forest plots. Variables were used for model development if there was no evidence of substantial heterogeneity. These candidate predictors were subsequently included in a multivariable logistic regression (“full model”). Restricted cubic splines restricted to three knots were used to evaluate whether transformation of continuous variables was appropriate. Variables in the final model were selected using stepwise backward selection using Akaike's information criterion ( *p* < 0.157). 25 Discrimination of the model was evaluated by calculating the c-statistic. The c-statistic can be calculated by using all possible pairs of patients where one patient experienced VTE and the other patient did not. The c-statistic is the proportion of such pairs in which the patient with VTE had a higher predicted probability of experiencing VTE than the subject who did not have VTE. Calibration was assessed by calculating the ratio between the number of observed and expected events (O:E ratio) and a calibration plot in each study. Ideally, the O:E ratio should be 1. If the OE ratio is <1, the model overestimates the probability of having recurrent VTE. If the O:E ratio is >1, the model underestimates the probability of having recurrent VTE. The model was validated using internal–external cross-validation, in which a new model was iteratively derived in *n* − 1 studies and subsequently evaluated in the remaining study. Performance measures were pooled across the internal–external cross-validation iterations by a random-effects meta-analysis with restricted maximum likelihood estimation and the Hartung–Knapp–Sidik–Jonkman method to calculate 95% confidence intervals (CIs). 26 Prediction intervals were calculated as a measure of between-study heterogeneity, which indicates expected model performance when the prediction model is applied within a specific study. All analyses were performed using R, version 2.2.1 ( www.R-project.org ).

## Results

### Characteristics of Study Group

Data from Hokusai VTE Cancer ( *n* = 1,046), SELECT-D ( *n* = 406), CLOT ( *n* = 676), and CATCH ( *n* = 914) were used (see Supplementary Table S2 (available in the online version) for study details). These trials enrolled patients from North-America, Europe, and Oceania. After exclusion of patients treated with vitamin K antagonists from CLOT and CATCH, the combined IPD set comprised 2,245 patients. The mean age was 63 years (standard deviation: 12) and 51% were female ( Table 1 ). The most frequent cancer types were colorectal (17%), lung (13%), and breast cancer (12%; Supplementary Table S3 , available in the online version). At randomization, 1,300 patients (59%) had metastatic cancer. Patients were randomly allocated to edoxaban (23%), rivaroxaban (9%), dalteparin (47%), or tinzaparin (20%). During 6 months of follow-up, 150 (6.7%) patients developed on-treatment recurrent VTE including PE with or without DVT (54%), DVT only (45%), or other VTE (1%), and 30.4% died ( Table 1 ).

**Table 1:** Baseline characteristics stratified by study

Demographics	Overall ( *n* = 2,245)	CATCH 14 ( *n* = 455)	CLOT 15 ( *n* = 338)	Hokusai 2 ( *n* = 1,046)	Select-D 3 ( *n* = 406)
Mean age, years (SD)	63.4 (11.8)	60.2 (12.9)	62.4 (11.7)	64.0 (11.3)	66.2 (10.6)
Male sex, *n* (%)	1,102 (49.1)	189 (41.5)	159 (47.0)	540 (51.7)	214 (52.7)
Mean weight, kg (SD)	75.6 (18.0)	67.2 (17.2)	73.6 (15.5)	78.9 (18.0)	78.4 (17.4)
ECOG performance score, *n* (%) a					
0	591 (26.5)	88 (19.4)	80 (23.7)	303 (29.2)	120 (30.0)
1	1,066 (47.8)	257 (56.6)	135 (39.9)	489 (47.1)	185 (46.2)
2	569 (25.5)	109 (24.0)	118 (34.9)	247 (23.8)	95 (23.8)
3	5 (0.2)	0 (0.0)	5 (1.5)	0 (0.0)	0 (0.0)
Li cancer type risk classification, *n* (%) b					
Very high-risk	298 (13.3)	60 (13.2)	18 (5.3)	143 (13.7)	77 (19.1)
High-risk	691 (30.8)	142 (31.2)	79 (23.4)	362 (34.6)	108 (26.7)
Intermediate-risk	385 (17.2)	68 (14.9)	52 (15.4)	162 (15.5)	103 (25.5)
Low-risk	867 (38.6)	185 (40.7)	187 (55.3)	379 (36.2)	116 (28.7)
Hematological cancer, *n* (%)	226 (10.1)	44 (9.7)	38 (11.2)	111 (10.6)	33 (8.2)
Metastatic disease, *n* (%)	1,300 (58.8)	250 (54.9)	223 (66.0)	595 (58.2)	232 (58.6)
Use of antiplatelets, *n* (%)	177 (8.0)	46 (10.1)	54 (16.0)	44 (4.3)	33 (8.1)
Platelet count >350 × 10 ^9^ /L, *n* (%)	371 (16.6)	102 (22.6)	73 (22.0)	126 (12.1)	70 (17.2)
Index event, *n* (%)					
PE ± DVT	1,209 (54%)	195 (42.9%)	103 (30.5%)	657 (62.8%)	295 (72.6%)
DVT only	1,036 (46%)	257 (56.0%)	235 (69.5%)	389 (37.2%)	111 (27.4%)
VTE treatment, *n*					
Edoxaban	522 (23.3)	0	0	522	0
Rivaroxaban	203 (9.0)	0	0	0	203
Dalteparin	1,065 (47.4)	0	338	524	203
Tinzaparin	455 (20.3)	455	0	0	0
Recurrent VTE on treatment, *n* (%)	150 (6.7)	31 (6.8)	27 (8.0)	66 (6.3)	26 (6.4)
Recurrent VTE type, *n* (%)					
PE ± DVT	81 (54.0)	20 (64.5)	13 (48.1)	35 (53.0)	13 (50.0)
DVT	67 (44.7)	11 (35.5)	14 (51.9)	31 (47.0)	11 (42.3)
Other	2 (<0.1)	0 (0.0)	0 (0.0)	0 (0.0)	2 (7.7)
All-cause mortality	925 (30.4%)	150 (33.4%)	130 (38.5%)	267 (25.5%)	104 (25.6%)

Abbreviations: DVT, deep vein thrombosis; ECOG, Eastern Cooperative Oncology Group; PE, Pulmonary embolism; SD, standard deviation; VTE, venous thromboembolism.

a 14 patients had missing data on ECOG performance status score.

b Very high-risk cancer types: pancreatic, gastroesophageal, bile duct, and gall bladder cancer; high-risk cancer types: lung, ovarian, uterine, bladder, kidney, testicular, primary brain cancer, aggressive non-Hodgkin lymphoma, multiple myeloma, and soft tissue sarcoma; intermediate-risk cancer type: colorectal cancer; low-risk cancers are all other cancer types. For two patients in the CLOT and two patients in the SELECT-D trial, data on cancer type was missing.

### Candidate Predictors

Supplementary Fig. S1 (available in the online version) and Supplementary Table S4 (available in the online version) show the association between the 15 candidate predictors and recurrent VTE in each study. Table 2 shows the results from the univariable logistic regression model. The candidate predictors with the strongest association with recurrent VTE were DVT only at randomization (OR: 1.80; 95% CI: 1.29–2.52, *I* ^2^ = 0%), breast cancer (OR: 0.41; 95% CI: 0.20–0.84, *I* ^2^ = 0%), and treatment with a DOAC (OR: 0.57; 95% CI: 0.38–0.85, *I* ^2^ = 0%; Table 2 ).

**Table 2:** Univariable and multivariable odds ratios for prediction of on-treatment recurrent VTE

Model to predict on-treatment recurrent VTE	Univariable odds ratio (95% CI)	Multivariable odds ratio (95% CI)	*p* -Value multivariable odds ratios
Age 1 (restricted cubic spline)	0.98 (0.96–1.01)	0.99 (0.96–1.01)	0.22
Age 2 (restricted cubic spline)	0.98 (0.95–1.02)	0.98 (0.95–1.02)	0.31
Presence of metastasis	1.40 (0.85–2.30)	1.44 (1.01–2.05)	0.05
Breast cancer	0.41 (0.20–0.84)	0.42 (0.20–0.87)	0.02
Treatment with a DOAC	0.57 (0.38–0.85)	0.66 (0.44–0.98)	0.04
Index event is DVT only	1.80 (1.29–2.52)	1.72 (1.31–2.27)	<0.01
**Other candidate predictors excluded during backward selection**			
ECOG performance score 1 or 2	1.23 (0.83–1.83)	n.a.	n.a.
Male sex	1.13 (0.81–1.58)	n.a.	n.a.
Use of antiplatelets	0.80 (0.37–1.47)	n.a.	n.a.
Platelet count > 350 × 10 ^9^ /L	0.98 (0.62–1.54)	n.a.	n.a.
Weight in kg	1.01 (0.97–1.01)	n.a.	n.a.
Lung cancer	0.99 (0.60–1.62)	n.a.	n.a.
Hepatobiliary cancer	1.53 (0.89–2.63)	n.a.	n.a.
Gynecological cancer	1.39 (0.89–2.17)	n.a.	n.a.
Urogenital cancer excluding prostate cancer	1.29 (0.68–2.45)	n.a.	n.a.
Hematological cancer	0.76 (0.41–1.40)	n.a.	n.a.
Li cancer risk classification (reference = low risk)			
Very high risk	1.47 (0.90–2.40)	n.a.	n.a.
High risk	1.12 (0.75–1.68)	n.a.	n.a.
Intermediate risk	1.02 (0.62–1.68)	n.a.	n.a.

Abbreviations: CI, confidence interval; DOAC, direct oral anticoagulant; DVT, deep vein thrombosis; ECOG, Eastern Cooperative Oncology Group; VTE, venous thromboembolism.

### Prediction Model

**Fig. 1:**
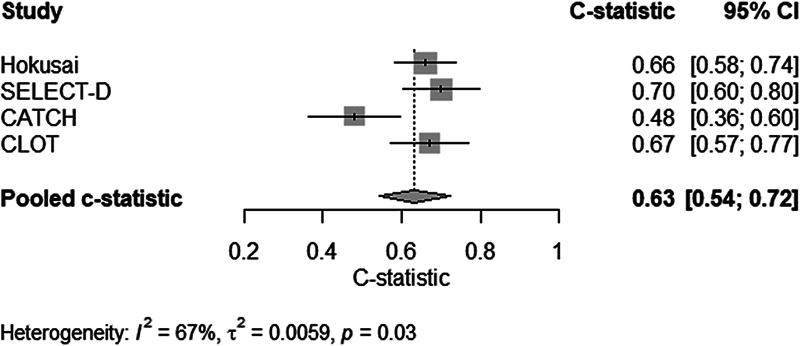
C-statistics and prediction interval in internal–external cross-validation.

**Fig. 2:**
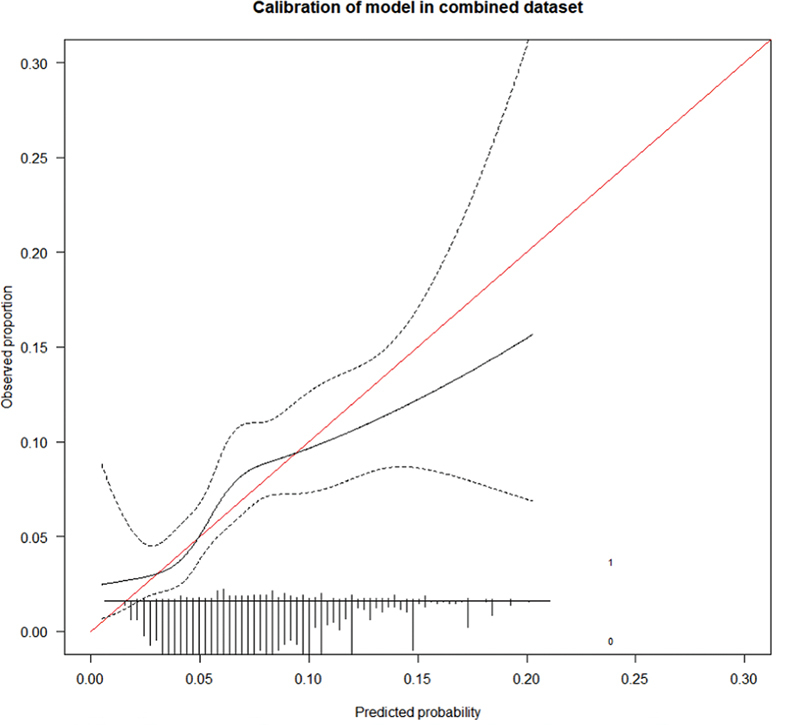
Calibration plot. Calibration in one imputed dataset is shown.

All candidate predictors were included in the full model. After stepwise backward selection, the following five predictors were retained in the final multivariable logistic regression model: age (continuous), breast cancer, metastatic disease, DOAC or LMWH treatment, and DVT only as an index event ( Table 2 ; formula provided in Supplementary Table S5 , available in the online version). The pooled apparent c-statistic of the model was 0.66 (95% CI: 0.61–0.70), which decreased to 0.63 (95% CI: 0.54–0.72; 95%, summary of confidence interval and prediction interval: 0.22–0.91) after internal–external cross-validation ( Fig. 1 ). Calibration-in-the-large was good with a ratio between observed and expected outcomes of 1.01 (95% CI: 0.85–1.21; Fig. 2 ). Calibration across the studies varied though ( Supplementary Fig. S2 , available in the online version), with poor calibration in the CLOT and CATCH trials and better calibration in the Hokusai VTE Cancer and SELECT-D. Specifically, the model underestimated recurrent VTE risk in SELECT-D trial and overestimated the risk in the CATCH trial.

## Discussion

Using IPD from four RCTs including more than 2,000 patients with cancer and acute VTE, five clinical predictors of recurrent on-treatment VTE were identified. The strongest predictors were DVT only (OR: 1.80), breast cancer (OR: 0.41), and treatment with a DOAC compared to LMWH (OR: 0.57). The clinical prediction model for the 6-month risk of on-treatment recurrent VTE including these five predictors had modest discrimination (c-statistic 0.63 after internal–external cross-validation) and calibration was inconsistent.

The Ottawa risk score is currently the only validated tool for assessment of the risk of recurrence after cancer-associated VTE. 11 The score's items include sex, previous VTE, cancer stage, and cancer type (breast or lung cancer). Two versions of the score have been developed: the original score that classifies patients as low or high risk, while the modified Ottawa score also includes an intermediate-risk group. Unfortunately, we were not able to formally evaluate the performance of the Ottawa scores since data on TNM classification were not collected in all RCTs. A systematic review and meta-analysis demonstrated that discrimination of the original (c-statistic 0.7; 95% CI: 0.6−0.8) and modified Ottawa scores (c-statistic 0.5; 95% CI: 0.5–0.6) is comparable to that of the clinical prediction model presented here. 11

Another prediction model for cancer-associated recurrent VTE was recently developed using Spanish electronic health record data from 16,407 cancer patients. 27 After feature selection and model training using machine learning, the items included in the model were age, previous VTE, VTE type, metastasis, adenocarcinoma, hemoglobin and serum creatinine levels, and platelet and leukocyte count. Discrimination of the model was also modest, with c-statistics ranging between 0.66 and 0.69 depending on the statistical technique used. Although this retrospective derivation study was well-powered, it is unclear how many events occurred during anticoagulant treatment and what the positive predictive value of the administrative codes used for recurrent VTE was. The model has not been externally validated yet. Unfortunately, we were also unable to validate this model due to missing information in our dataset, in particular several laboratory data were not available.

Tumor type is by far the strongest predictor for a first episode of cancer-associated VTE, but the prognostic value of tumor type for recurrent VTE is less clear. 28 A large Danish population-based cohort including 34,072 patients with cancer and a first VTE diagnosis identified cancer type as a predictor for recurrent VTE, but the associations were generally weak. 29 The strongest association was observed for genitourinary (subdistribution hazard ratio [HR]: 1.35; 95% CI: 1.06–1.71) and lung cancer (subdistribution HR: 1.26; 95% CI: 1.03–1.53). In the present study, only breast cancer was retained as a protective risk factor in the final model for recurrent VTE. Discrimination was not improved when the validated tumor risk classification for a first VTE proposed by Li and colleagues was used. 23 Similarly, cancer type was not retained in the aforementioned model by Muñoz and colleagues. These findings suggest that the association between cancer type and a first VTE is stronger than that with a recurrent VTE, a similar phenomenon previously observed for hereditary thrombophilia that has been attributed to collider bias. 30 Whether a specific cancer type risk classification for recurrent VTE improves prediction needs further study.

The current study had several strengths. We were able to obtain high quality patient-level data from the four open-label RCTs that were reasonably homogeneous in design and outcome definitions. The proportion of missing data was low, few patients were lost to follow-up, and all recurrent thromboembolic events were adjudicated. The number of outcome events per variable included in the full model was about 27, which is generally believed to be sufficient for model development. The internal–external cross-validation procedure allowed us to validate the model using all available data unlike a split-sample approach.

Some limitations merit consideration. First, we were not able to assess other potential predictors of recurrent VTE, such as cancer stage, kidney function, hemoglobin levels, leukocyte count, history of VTE, and cancer treatment, as they were missing in one or more studies. Platelet count had to be used dichotomously because continuous data were not available in all studies. Second, we could not directly compare the performance of the present model to other previously developed risk assessment tools such as the Ottawa score, because of missing predictors in our database. Third, we did not have access to data from more recent trials, such as CANVAS or Caravaggio. 5 6 Fourth, we only used data from RCTs which can limit generalizability. The strict eligibility criteria used in the clinical trials likely resulted in patients with a better prognosis than in the general population, with unclear potential effect on the performance of the model. External validation of the model in other settings would be needed. Fifth, participants in CATCH and CLOT were enrolled more than 10 to 20 years ago respectively, with resulting differences in cancer treatment, follow-up (e.g., staging scans), and diagnostic procedures for VTE compared with the Hokusai VTE cancer and SELECT-D trials. Also, there was some variation in the definition of recurrent VTE across the trials. In CLOT, incidental VTE was not considered in the primary outcome. Hokusai VTE cancer and CATCH adjudicated unexplained death as fatal PE, since PE could not be ruled out. These differences may have led to the poor calibration observed in the CATCH and CLOT trials. Furthermore, the discriminatory ability of the final model was lower in the CATCH trial compared with the other three trials, which might be explained by differences in case mix (e.g., differences in cancer type with other recurrent VTE rates), differences in treatment (e.g., full-dose LMWH in CATCH control group compared to maintenance-dose LMWH in the other trials), differences in outcome definition (about half of recurrent VTE in CATCH were deaths for which PE could not be ruled out), or just chance.

Discrimination of the present prediction model for recurrent VTE was not better than that of the (modified) Ottawa score nor the model by Muñoz et al. 11 27 Discrimination of all these models is modest at best (c-statistics ≤ 0.70), but comparable to performance of a prediction model for recurrent VTE in the general population. 31 Prediction of recurrent VTE is challenging because it is often provoked by factors that occur during anticoagulant treatment, such as surgery, changes in systemic anticancer therapy, hospitalization for an acute medical illness, or cancer progression. Other contributing factors include interruptions of anticoagulation for surgery or bleeding and adherence, which may be lower for LMWH than for DOACs. Such factors cannot be incorporated in statistical prediction models that are applied only once at baseline. Dynamic prediction models can overcome this limitation by allowing periodic reassessment, but they are much harder to develop and validate. Extending the clinical model with plasma biomarkers, such as soluble P-selectin, may improve prediction at start of anticoagulation at the cost of adding complexity. 32

Another important point is the timing of applying a prediction model to guide treatment decisions. Patients classified as being at high risk of recurrent at the index VTE should probably not have a LMWH dose reduction at 1 month, but it is less clear if such patients should also continue full-dose anticoagulation beyond 3 to 6 months. Ideally, a new assessment at 3 to 6 months is needed to guide this decision, which is of particular interest given the upcoming studies that evaluate a low-dose DOAC for secondary prevention in cancer patients, such as the API-CAT trial (NCT03692065) and EVE trial, as well as trials evaluating factor XI inhibitors. 33 Accurate prediction of recurrent VTE at different time points during the course of the disease remains an important unmet need.

In conclusion, we have developed a prediction model with five predictors using the IPD of four RCTs. However, discrimination of the final clinical prediction model was modest, indicating that prediction of cancer-associated recurrent VTE at diagnosis of acute VTE remains challenging and that other contributing factors need to be identified.

## Notes

**What is known about this topic?**

- Recurrent on-treatment venous thromboembolism is a common complication of cancer-associated thrombosis.
- The Ottawa scores are validated scores for prediction of recurrent cancer-associated thrombosis, but the use of the scores in clinical practice is limited due to modest discriminatory ability.

**What does this paper add?**

- This IPD meta-analysis of four large randomized controlled trials identified clinical predictors for recurrent cancer-associated thrombosis before start of anticoagulant treatment.
- We derived a clinical prediction model based on age, breast cancer, metastatic disease, treatment with a DOAC, and DVT only as index events.
- The model only had modest discriminatory performance, highlighting the need for new risk assessment tools for recurrent cancer-associated thrombosis during treatment.
