# Supplementary material for: Risk of Recurrent Venous Thromboembolism in Patients with Cancer: An Individual Patient Data Meta-analysis and Development of a Prediction Model
Source: Thromb Haemost. 2024 Oct 16;125(6):589–96. doi: 10.1055/a-2418-3960 (PMC12115544; doi:10.1055/a-2418-3960)
Supplement: Supplementary file 1 — Supplementary Material [file 10-1055-a-2418-3960-s24070338.pdf]

Supplementary Table S1 TRIPOD criteria checklist

Section/topic	Item	Checklist items	
**Title and abstract**			**Page**
Title	1	Identify the study as developing and/or validating a multivariable prediction model, the target population, and the outcome to be predicted.	1
Abstract	2	Provide a summary of objectives, study design, setting, participants, sample size, predictors, outcome, statistical analysis, results, and conclusions.	3
**Introduction**			
Background and objectives	3a	Explain the medical context (including whether diagnostic or prognostic) and rationale for developing or validating the multivariable prediction model, including references to existing models.	6
	3b	Specify the objectives, including whether the study describes the development or validation of the model or both.	6
**Methods**			
Source of data	4a	Describe the study design or source of data (e.g., randomized trial, cohort, or registry data), separately for the development and validation data sets, if applicable.	7
	4b	Specify the key study dates, including start of accrual; end of accrual; and, if applicable, end of follow-up.	7
Participants	5a	Specify key elements of the study setting (e.g., primary care, secondary care, general population) including number and location of centers.	7
	5b	Describe eligibility criteria for participants.	7
	5c	Give details of treatments received, if relevant.	7
Outcome	6a	Clearly define the outcome that is predicted by the prediction model, including how and when assessed.	7
	6b	Report any actions to blind assessment of the outcome to be predicted.	7
Predictors	7a	Clearly define all predictors used in developing or validating the multivariable prediction model, including how and when they were measured.	8
	7b	Report any actions to blind assessment of predictors for the outcome and other predictors.	8
Sample size	8	Explain how the study size was arrived at.	7
Missing data	9	Describe how missing data were handled (e.g., complete-case analysis, single imputation, multiple imputation) with details of any imputation method.	8
Statistical analysis methods	10a	Describe how predictors were handled in the analyses.	9
	10b	Specify type of model, all model-building procedures (including any predictor selection), and method for internal validation.	8–9
	10d	Specify all measures used to assess model performance and, if relevant, to compare multiple models.	9
Risk groups	11	Provide details on how risk groups were created, if done.	8–9
**Results**			
Participants	13a	Describe the flow of participants through the study, including the number of participants with and without the outcome and, if applicable, a summary of the follow-up time. A diagram may be helpful.	10
	13b	Describe the characteristics of the participants (basic demographics, clinical features, available predictors), including the number of participants with missing data for predictors and outcome.	10
Model development	14a	Specify the number of participants and outcome events in each analysis.	10
	14b	If done, report the unadjusted association between each candidate predictor and outcome.	11
Model specification	15a	Present the full prediction model to allow predictions for individuals (i.e., all regression coefficients, and model intercept or baseline survival at a given time point).	11

(*Continued*)

 © 2024. The Author(s).

Supplementary Table S1 (*Continued*)

Section/topic	Item	Checklist items	
	15b	Explain how to the use the prediction model.	11
Model performance	16	Report performance measures (with Cis) for the prediction model.	11
**Discussion**			
Limitations	18	Discuss any limitations of the study (such as nonrepresentative sample, few events per predictor, missing data).	13–14
Interpretation	19b	Give an overall interpretation of the results, considering objectives, limitations, and results from similar studies, and other relevant evidence.	11–14
Implications	20	Discuss the potential clinical use of the model and implications for future research.	14
**Other information**			
Supplementary information	21	Provide information about the availability of supplementary resources, such as study protocol, Web calculator, and data sets.	N.A
Funding	22	Give the source of funding and the role of the funders for the present study.	1

Abbreviation: CI, confidence interval.
Note: We recommend using the TRIPOD Checklist in conjunction with the TRIPOD Explanation and Elaboration document.

 © 2024. The Author(s).

Supplementary Table S2 Studies eligible for inclusion

Study	Study period	Intervention	Control	Efficacy outcome	Follow-up	Patients (*n*)	Recurrences, *n* (%)	Lost to follow-up
CLOT	1999–2001	Dalteparin 200 IU/kg od	VKA + dalteparin for first 5–7 days	Symptomatic VTE	6 months	676	80 (11.8%)	Not reported
CATCH	2010–2013	Tinzaparin 175 IU/kg od	Warfarin + tinzaparin 175 IU/kg od for first 5–10 days	Symptomatic VTE	6 months	914	76 (8.4%)	14
Hokusai-VTE Cancer	2015–2016	Edoxaban 60 mg or 30 mg od	Dalteparin 200 IU/kg od for first 30 days followed by 150 IU/kg od	Symptomatic or incidental VTE	12 months	1,046	80 (7.6%)[a]	8
SELECT-D	2013–2016	Rivaroxaban 15 mg bid for first 21 days followed by 20 mg od	Dalteparin 200 IU/kg od for first 30 days followed by 150 IU/kg od	Symptomatic or incidental VTE	6 months	406	26 9 (6.4%)	1
**TOTAL**						**3.042**	**262 (8.6%)**	**23**

Abbreviations: LMWH, low-molecular-weight heparin; VKA, vitamin K antagonist; VTE, venous thromboembolism.
[a]Number of events presented are during the first 6 months of the study period.

 © 2024. The Author(s).

Supplementary Table S3 Cancer types in per study and in combined dataset

	Overall	CATCH[14]	CLOT[15]	Hokusai[2]	Select-D[3]
Total number of patients	2,245	455	338	1,046	406
Cancer type, *n* (%)[a]					
Bladder	68 (3.0)	14 (3.1)	10 (3.0)	30 (2.9)	14 (3.5)
Brain	49 (2.2)	11 (2.4)	14 (4.2)	21 (2.0)	3 (0.7)
Breast	262 (11.7)	37 (8.1)	59 (7.6)	125 (12.0)	41 (10.1)
Cervix	74 (3.3)	46 (10.1)	14 (4.2)	14 (1.3)	0 (0.0)
Colorectal	385 (17.2)	68 (14.9)	52 (15.5)	162 (15.5)	103 (25.5)
Endometrium	55 (2.5)	18 (4.0)	0 (0.0)	37 (3.5)	0 (0.0)
Gallbladder	10 (0.4)	6 (1.3)	0 (0.0)	0 (0.0)	4 (1.0)
Gastro-esophageal	130 (5.8)	29 (6.4)	6 (1.8)	54 (5.2)	41 (10.1)
Head and neck	23 (1.0)	5 (1.1)	0 (0.0)	18 (1.7)	0 (0.0)
Hepatobiliary	37 (1.7)	9 (2.0)	0 (0.0)	26 (2.5)	2 (0.5)
Leukemia	37 (1.7)	4 (0.9)	8 (2.4)	19 (1.8)	6 (1.5)
Lung	287 (12.8)	48 (10.5)	40 (11.9)	152 (14.5)	47 (11.6)
Lymphoma	118 (5.3)	26 (5.7)	26 (7.7)	44 (4.2)	22 (5.4)
Melanoma	20 (0.9)	5 (1.1)	0 (0.0)	15 (1.4)	0 (0.0)
Multiple myeloma	63 (2.8)	14 (3.1)	4 (1.2)	40 (3.8)	5 (1.2)
Ovarian	124 (5.5)	31 (6.8)	11 (3.3)	52 (5.0)	30 (7.4)
Pancreas	121 (5.4)	16 (3.5)	12 (3.6)	63 (6.0)	30 (7.4)
Prostate	129 (5.8)	21 (4.6)	25 (7.4)	62 (5.9)	21 (5.2)
Renal	36 (1.6)	3 (0.7)	0 (0.0)	26 (2.5)	7 (1.7)
Sarcoma	36 (1.6)	8 (1.8)	0 (0.0)	26 (2.5)	2 (0.5)
Testicular	28 (1.2)	13 (2.9)	0 (0.0)	15 (1.4)	0 (0.0)
Unknown primary	23 (1.0)	3 (0.7)	5 (1.5)	9 (0.9)	6 (1.5)
Other gastrointestinal	1 (0.0)	1 (0.2)	0 (0.0)	0 (0.0)	0 (0.0)
Other gynecological	36 (1.6)	10 (2.2)	13 (3.9)	0 (0.0)	13 (3.2)
Other solid	81 (3.6)	9 (2.0)	37 (11.0)	28 (2.7)	7 (1.7)
Other hematological	8 (0.4)	0 (0.0)	0 (0.0)	8 (0.8)	0 (0.0)
Li cancer classification, *n* (%)[a]					
Very high risk	298 (13.3)	60 (13.2)	18 (5.3)	143 (13.7)	77 (19.1)
High risk	691 (30.8)	142 (31.2)	79 (23.4)	362 (34.6)	108 (26.7)
Intermediate risk	385 (17.2)	68 (14.9)	52 (15.4)	162 (15.5)	103 (25.5)
Low risk	867 (38.6)	185 (40.7)	187 (55.3)	379 (36.2)	116 (28.7)

[a]For 2 patients in the CLOT and 2 patients in the SELECT-D trial, data on cancer type were missing.

 © 2024. The Author(s).

Supplementary Table S4 Crude and adjusted hazard ratios for on-treatment recurrent VTE in original studies

Table S4A Crude hazard ratios for 6-month risk of on-treatment recurrent VTE including all patients (including vitamin K antagonists users)

Predictor	Hokusai				Select-D				CATCH				CLOT			
	HR	Lower CI	Upper CI	*p*-Value	HR	Lower CI	Upper CI	*p*-Value	HR	Lower CI	Upper CI	*p*-Value	HR	Lower CI	Upper CI	*p*-Value
Age	0.98	0.96	1.00	0.03	0.99	0.96	1.01	0.31	0.98	0.97	1.00	0.05	0.97	0.96	0.99	<0.001
Weight	1.01	0.99	1.02	0.36	1.01	0.99	1.04	0.42	0.99	0.97	1.00	0.13	1.00	0.99	1.01	0.85
Male sex	1.05	0.65	1.70	0.84	1.45	0.66	3.20	0.35	1.14	0.70	1.85	0.59	1.64	1.05	2.57	0.03
ECOG performance score (reference is score of 0)																
ECOG 1	1.20	0.68	2.13	0.52	2.83	0.95	8.41	0.06	1.99	0.89	4.44	0.09	1.11	0.59	2.09	0.75
ECOG 2	1.26	0.63	2.52	0.51	2.08	0.58	7.49	0.26	3.83	1.69	8.68	<0.001	1.87	1.02	3.43	0.04
Use of antiplatelets	0.37	0.05	2.68	0.32	1.01	0.24	4.36	0.98	0.25	0.06	1.02	0.05	1.47	0.87	2.48	0.15
Platelets >350	1.36	0.70	2.67	0.37	0.98	0.34	2.82	0.97	0.89	0.50	1.59	0.70	0.99	0.56	1.74	0.98
Index event DVT	1.86	1.12	3.08	0.02	2.85	1.32	6.14	0.01	1.36	0.74	2.49	0.33	1.16	0.64	2.11	0.62
Metastatic Cancer	1.88	1.11	3.20	0.02	1.87	0.81	4.34	0.14	1.66	1.00	2.76	0.05	3.16	1.71	5.86	<0.001
GU or GI cancer	1.33	0.82	2.15	0.25	1.11	0.51	2.41	0.79	1.34	0.83	2.18	0.23	0.85	0.53	1.37	0.51
Hepatobiliary cancers	2.03	1.01	4.06	0.05	2.71	0.94	7.85	0.07	3.07	1.51	6.24	<0.01	2.56	1.11	5.93	0.03
Genitourinary cancers	1.27	0.69	2.33	0.44	1.17	0.45	3.01	0.75	1.94	1.19	3.15	<0.01	0.76	0.38	1.52	0.44
Breast cancer	0.53	0.21	1.33	0.18	0.33	0.04	2.45	0.28	n/e	n/e	n/e	n/e	0.25	0.09	0.69	<0.01
Lung cancer	1.22	0.62	2.39	0.57	0.74	0.18	3.14	0.68	1.12	0.54	2.32	0.77	3.14	1.94	5.07	<0.001
Upper GI cancers	1.61	0.39	6.61	0.51	0.77	0.18	3.27	0.73	1.64	0.65	4.11	0.29	1.32	0.34	5.12	0.69
Prostate cancer	0.23	0.03	1.66	0.14	1.36	0.32	5.78	0.67	1.89	0.76	4.74	0.17	0.64	0.23	1.74	0.38
Urological cancers	1.10	0.44	2.70	0.84	1.40	0.35	5.70	0.64	1.64	0.77	3.50	0.20	1.15	0.43	3.10	0.78
Gynecological cancers	1.36	0.65	2.85	0.42	1.02	0.31	3.30	0.98	1.77	1.06	2.94	0.03	0.61	0.24	1.50	0.28
Pancreatic cancer	1.53	0.63	3.74	0.35	3.45	1.19	9.99	0.02	2.39	0.84	6.82	0.10	2.56	1.11	5.93	0.03
Li cancer classification (reference is low risk)																
Very high risk cancer	2.79	1.36	5.74	0.01	1.13	0.41	3.11	0.81	2.35	1.22	4.52	0.01	2.64	1.22	5.70	0.01
High risk cancer	0.58	0.31	1.09	0.09	1.74	0.60	5.06	0.31	0.68	0.39	1.19	0.18	0.49	0.30	0.79	<0.001
Intermediate risk cancer	0.55	0.26	1.17	0.12	1.83	0.63	5.36	0.27	2.64	0.80	8.75	0.11	1.24	0.57	2.68	0.59

Abbreviations: CI, confidence interval; DOAC, direct oral anticoagulant; DVT, deep vein thrombosis; ECOG, Eastern Cooperative Oncology Group; GI, gastrointestinal; GU, genitourinary; HR, hazard ratio; VTE, venous thromboembolism.

 © 2024. The Author(s).

Table S4B Age and sex adjusted hazard ratios for 6-month risk of on-treatment recurrent VTE (including vitamin K antagonists users)

Predictor	Hokusai				Select-D				CATCH				CLOT			
	HR	Lower CI	Upper CI	*p*-Value	HR	Lower CI	Upper CI	*p*-Value	HR	Lower CI	Upper CI	*p*-Value	HR	Lower CI	Upper CI	*p*-Value
Weight	1.00	0.99	1.02	0.69	1.01	0.98	1.04	0.64	0.99	0.97	1.00	0.14	0.99	0.98	1.00	0.20
ECOG performance score (reference is score of 0)																
ECOG 1	1.22	0.68	2.18	0.50	3.11	1.07	9.05	0.04	2.09	0.94	4.67	0.07	1.28	0.67	2.44	0.45
ECOG 2	1.36	0.67	2.75	0.39	2.48	0.74	8.34	0.14	3.66	1.59	8.43	<0.001	2.03	1.10	3.73	0.02
Use of antiplatelets	0.40	0.05	2.90	0.36	1.10	0.24	5.10	0.90	0.27	0.07	1.13	0.07	1.52	0.91	2.54	0.11
Platelets >350	1.33	0.68	2.60	0.41	1.01	0.35	2.89	0.98	0.86	0.47	1.55	0.61	0.94	0.53	1.67	0.83
Index event DVT only	2.18	1.34	3.55	0.00	3.29	1.53	7.08	0.00	0.89	0.55	1.46	0.65	1.25	0.76	2.06	0.37
Metastatic cancer	1.84	1.09	3.12	0.02	1.83	0.80	4.23	0.16	1.65	0.99	2.74	0.05	3.54	1.92	6.51	<0.001
GU or GI cancer	1.40	0.82	2.40	0.21	1.00	0.44	2.28	1.00	1.39	0.85	2.29	0.19	0.86	0.50	1.46	0.58
Hepatobiliary cancers	2.06	1.02	4.15	0.04	2.73	0.97	7.74	0.06	3.17	1.56	6.41	<0.01	2.54	1.07	6.05	0.03
Genitourinary cancers	1.21	0.67	2.21	0.53	1.27	0.47	3.40	0.64	1.99	1.16	3.42	0.01	0.87	0.42	1.79	0.70
Breast cancer	0.49	0.19	1.27	0.14	0.36	0.04	2.91	0.34	n/e	n/e	n/e	n/e	0.28	0.10	0.83	0.02
Lung cancer	1.26	0.64	2.48	0.50	0.69	0.16	3.00	0.62	1.11	0.53	2.36	0.78	2.89	1.78	4.69	<0.001
Upper GI cancers	1.69	0.42	6.87	0.46	0.77	0.18	3.22	0.72	1.63	0.64	4.14	0.30	1.30	0.35	4.78	0.69
Prostate cancer	0.24	0.03	1.75	0.16	1.29	0.29	5.66	0.74	2.36	0.87	6.43	0.09	0.59	0.21	1.64	0.31
Urological cancers	0.99	0.38	2.54	0.98	1.22	0.30	4.97	0.78	1.34	0.57	3.13	0.50	1.19	0.44	3.26	0.73
Gynecological cancers	1.42	0.64	3.16	0.39	1.29	0.34	4.88	0.70	2.33	1.19	4.56	0.01	0.69	0.26	1.81	0.45
Pancreatic cancer	1.58	0.64	3.91	0.32	3.46	1.24	9.65	0.02	2.42	0.86	6.85	0.10	2.54	1.07	6.05	0.03
Li cancer classification (reference is low risk)																
Very high risk cancer	3.27	1.61	6.63	<0.001	0.95	0.36	2.50	0.91	2.18	1.10	4.31	0.03	2.49	1.11	5.55	0.03
High risk cancer	0.57	0.31	1.05	0.07	1.85	0.64	5.36	0.25	0.69	0.37	1.27	0.23	0.50	0.30	0.84	0.01
Intermediate risk cancer	0.48	0.23	0.99	0.05	1.77	0.53	5.94	0.35	2.58	0.68	9.76	0.16	1.24	0.56	2.75	0.60

Abbreviations: CI, confidence interval; DOAC, direct oral anticoagulant; DVT, deep vein thrombosis; ECOG, Eastern Cooperative Oncology Group; GI, gastrointestinal; GU, genitourinary; HR, hazard ratio; n/e, not estimable; VTE, venous thromboembolism.

 © 2024. The Author(s).

Table S4C Model-adjusted hazard ratios for 6-month risk of on-treatment recurrent VTE based on all included patients in original studies (including vitamin K antagonists users); this model included the following predictors after backward selection: age, sex, ECOG performance score, index event DVT, metastatic disease, Li cancer classification

Predictor	Hokusai				Select-D				CATCH				CLOT			
	HR	Lower CI	Upper CI	*p*-Value	HR	Lower CI	Upper CI	*p*-Value	HR	Lower CI	Upper CI	*p*-Value	HR	Lower CI	Upper CI	*p*-Value
Age 1	0.98	0.95	1.02	0.42	1.03	0.96	1.11	0.42	0.99	0.96	1.02	0.41	0.96	0.93	1.00	0.04
Age 2	0.98	0.94	1.03	0.44	0.91	0.82	1.02	0.10	1.00	0.95	1.06	0.94	1.01	0.96	1.06	0.81
Male sex	0.84	0.51	1.39	0.49	1.64	0.70	3.85	0.25	1.11	0.64	1.91	0.72	1.78	1.12	2.84	0.01
ECOG performance score (reference is ECOG score 0)																
ECOG 1	1.37	0.76	2.48	0.29	2.90	1.01	8.32	0.05	1.83	0.82	4.09	0.14	0.94	0.50	1.76	0.84
ECOG 2	1.24	0.60	2.53	0.56	2.61	0.71	9.52	0.15	3.21	1.35	7.62	0.01	1.42	0.76	2.67	0.27
Index event DVT only	1.98	1.17	3.34	0.01	3.12	1.42	6.86	<0.001	1.45	0.79	2.68	0.23	1.12	0.60	2.09	0.73
Metastatic cancer	1.67	0.99	2.83	0.06	2.40	0.97	5.93	0.06	1.43	0.82	2.49	0.21	3.43	1.80	6.54	<0.001
Li cancer classification (reference is low risk)																
Very high risk cancer	2.61	1.26	5.39	0.01	1.14	0.42	3.07	0.80	2.17	1.04	4.50	0.04	1.99	0.88	4.52	0.10
High risk cancer	1.67	0.89	3.12	0.11	0.56	0.19	1.60	0.28	1.34	0.72	2.49	0.35	1.95	1.15	3.31	0.01
Intermediate risk cancer	1.75	0.82	3.73	0.15	0.43	0.12	1.58	0.20	0.38	0.10	1.38	0.14	0.72	0.32	1.59	0.41

Abbreviations: CI, confidence interval; DOAC, direct oral anticoagulant; DVT, deep vein thrombosis; ECOG, Eastern Cooperative Oncology Group; GI, gastrointestinal; GU, genitourinary; HR, hazard ratio; n/e, not estimable; VTE, venous thromboembolism.

 © 2024. The Author(s).

Table S4D Crude hazard ratios for 6-month risk of on-treatment recurrent VTE excluding patients using vitamin K antagonist

Predictor	Hokusai				Select-D				CATCH				CLOT			
	HR	Lower CI	Upper CI	*p*-Value	HR	Lower CI	Upper CI	*p*-Value	HR	Lower CI	Upper CI	*p*-Value	HR	Lower CI	Upper CI	*p*-Value
Age	0.98	0.96	1.00	0.03	0.99	0.96	1.01	0.31	0.98	0.95	1.00	0.07	0.97	0.94	0.99	0.01
Weight	1.01	0.99	1.02	0.36	1.01	0.99	1.04	0.42	0.99	0.96	1.01	0.33	1.01	0.99	1.03	0.37
Male sex	1.05	0.65	1.70	0.84	1.45	0.66	3.20	0.35	0.87	0.42	1.79	0.71	1.40	0.66	2.98	0.38
ECOG performance score (reference is ECOG score 0)																
ECOG 1	1.20	0.68	2.13	0.52	2.83	0.95	8.41	0.06	1.09	0.40	2.99	0.87	0.73	0.26	1.99	0.53
ECOG 2	1.26	0.63	2.52	0.51	2.08	0.58	7.49	0.26	2.79	1.03	7.54	0.04	1.43	0.57	3.59	0.44
Use of antiplatelets	0.37	0.05	2.68	0.32	1.01	0.24	4.36	0.98	0.28	0.04	2.12	0.22	1.22	0.47	3.18	0.68
Platelets >350	1.36	0.70	2.67	0.37	0.98	0.34	2.82	0.97	0.53	0.19	1.50	0.23	1.33	0.56	3.17	0.52
Metastatic cancer	1.88	1.11	3.20	0.02	1.87	0.81	4.34	0.14	0.95	0.47	1.93	0.89	1.73	0.73	4.06	0.21
Index event DVT only	1.86	1.12	3.08	0.02	2.85	1.32	6.14	0.01	0.98	0.44	2.19	0.96	0.96	0.36	2.51	0.93
GU or GI cancer	1.33	0.82	2.15	0.25	1.11	0.51	2.41	0.79	1.26	0.62	2.56	0.52	0.90	0.40	2.05	0.80
Hepatobiliary cancers	2.03	1.01	4.06	0.05	2.71	0.94	7.85	0.07	1.79	0.53	6.01	0.35	n/e	n/e	n/e	n/e
Genitourinary cancers	1.27	0.69	2.33	0.44	1.17	0.45	3.01	0.75	2.26	1.12	4.57	0.02	0.78	0.23	2.58	0.68
Breast cancer	0.53	0.21	1.33	0.18	0.33	0.04	2.45	0.28	n/e	n/e	n/e	n/e	0.36	0.09	1.50	0.16
Lung cancer	1.22	0.62	2.39	0.57	0.74	0.18	3.14	0.68	0.63	0.15	2.59	0.52	1.92	0.73	5.06	0.19
Upper GI cancers	1.61	0.39	6.61	0.51	0.77	0.18	3.27	0.73	2.26	0.69	7.41	0.18	n/e	n/e	n/e	n/e
Urological cancers	1.10	0.44	2.70	0.84	1.40	0.35	5.70	0.64	1.40	0.44	4.41	0.57	1.17	0.17	8.31	0.87
Gynecological cancers	1.36	0.65	2.85	0.42	1.02	0.31	3.30	0.98	2.21	1.08	4.54	0.03	0.67	0.16	2.85	0.59
Pancreatic cancer	1.53	0.63	3.74	0.35	3.45	1.19	9.99	0.02	2.58	0.58	11.60	0.22	n/e	n/e	n/e	n/e
Li cancer classification (reference is low risk)																
Very high risk	2.79	1.36	5.74	0.01	1.13	0.41	3.11	0.81	1.65	0.64	4.24	0.30	0.00	0.00	0.00	0.00
High risk	0.58	0.31	1.09	0.09	1.74	0.60	5.06	0.31	1.05	0.47	2.37	0.90	0.63	0.26	1.52	0.31
Intermediate risk	0.55	0.26	1.17	0.12	1.83	0.63	5.36	0.27	5.52	0.73	42.00	0.10	0.60	0.23	1.58	0.30

Abbreviations: CI, confidence interval; DVT, deep vein thrombosis; ECOG, Eastern Cooperative Oncology Group; GI, gastrointestinal; GU, genitourinary; HR, hazard ratio; n/e, not estimable; VTE, venous thromboembolism.

 © 2024. The Author(s).

Table S4E Age- and sex-adjusted hazard ratios for 6-month risk of on-treatment recurrent VTE excluding patients using vitamin K antagonist

Predictor	Hokusai				Select-D				CATCH				CLOT			
	HR	Lower CI	Upper CI	*p*-Value	HR	Lower CI	Upper CI	*p*-Value	HR	Lower CI	Upper CI	*p*-Value	HR	Lower CI	Upper CI	*p*-Value
Weight	1.00	0.99	1.02	0.69	1.01	0.98	1.04	0.64	0.99	0.97	1.02	0.47	1.00	0.99	1.02	0.58
ECOG performance score (reference is ECOG score 0)																
ECOG 1	1.22	0.68	2.18	0.50	3.11	1.07	9.05	0.04	1.14	0.42	3.08	0.80	0.83	0.29	2.42	0.74
ECOG 2	1.36	0.67	2.75	0.39	2.48	0.74	8.34	0.14	2.63	0.93	7.42	0.07	1.66	0.63	4.41	0.31
Use of antiplatelets	0.40	0.05	2.90	0.36	1.10	0.24	5.10	0.90	0.33	0.05	2.41	0.28	1.32	0.51	3.44	0.57
Platelets >350	1.33	0.68	2.60	0.41	1.01	0.35	2.89	0.98	0.47	0.16	1.36	0.16	1.22	0.51	2.97	0.65
Index event DVT only	2.18	1.34	3.55	0.00	3.29	1.53	7.08	0.00	0.69	0.34	1.41	0.31	1.45	0.60	3.53	0.41
Metastatic cancer	1.84	1.09	3.12	0.02	1.83	0.80	4.23	0.16	0.92	0.45	1.87	0.81	2.05	0.90	4.67	0.09
GU or GI cancer	1.40	0.82	2.40	0.21	1.00	0.44	2.28	1.00	1.44	0.74	2.83	0.28	0.92	0.36	2.35	0.87
Hepatobiliary cancers	2.06	1.02	4.15	0.04	2.73	0.97	7.74	0.06	1.74	0.51	5.91	0.37	n/e	n/e	n/e	n/e
Genitourinary cancers	1.21	0.67	2.21	0.53	1.27	0.47	3.40	0.64	2.04	0.96	4.34	0.06	0.85	0.24	3.04	0.81
Breast cancer	0.49	0.19	1.27	0.14	0.36	0.04	2.91	0.34	n/e	n/e	n/e	n/e	0.38	0.07	1.93	0.24
Lung cancer	1.26	0.64	2.48	0.50	0.69	0.16	3.00	0.62	0.62	0.14	2.70	0.52	1.88	0.71	4.98	0.20
Upper GI cancers	1.69	0.42	6.87	0.46	0.77	0.18	3.22	0.72	2.28	0.70	7.41	0.17	n/e	n/e	n/e	n/e
Prostate cancer	0.24	0.03	1.75	0.16	1.29	0.29	5.66	0.74	6.08	1.79	20.72	0.00	0.42	0.06	3.14	0.40
Urological cancers	0.99	0.38	2.54	0.98	1.22	0.30	4.97	0.78	1.17	0.36	3.80	0.79	1.16	0.17	7.72	0.88
Gynecological cancers	1.42	0.64	3.16	0.39	1.29	0.34	4.88	0.70	2.55	0.99	6.55	0.05	0.74	0.16	3.51	0.70
Pancreatic cancer	1.58	0.64	3.91	0.32	3.46	1.24	9.65	0.02	2.50	0.56	11.18	0.23	n/e	n/e	n/e	n/e
Li cancer classification (reference is low risk)																
Very high risk	3.27	1.61	6.63	0.00	0.95	0.36	2.50	0.91	1.61	0.60	4.28	0.34	n/e	n/e	n/e	n/e
High risk	0.57	0.31	1.05	0.07	1.85	0.64	5.36	0.25	1.08	0.44	2.65	0.87	0.64	0.26	1.59	0.33
Intermediate risk	0.48	0.23	0.99	0.05	1.77	0.53	5.94	0.35	5.52	0.70	43.23	0.10	0.56	0.19	1.63	0.29

Abbreviations: CI, confidence interval; DVT, deep vein thrombosis; ECOG, Eastern Cooperative Oncology Group; GI, gastrointestinal; GU, genitourinary; HR, hazard ratio; n/e, not estimable; VTE, venous thromboembolism.

 © 2024. The Author(s).

Supplementary Table S5 Model for prediction of 6-month risk of on-treatment recurrent VTE

Predictors	β	Standard error	*p*-Value
Intercept	−1.90	0.66	<0.01
Age 1[a]	−0.01	0.01	0.22
Age 2[a]	−0.02	0.02	0.31
Metastatic disease	0.36	0.18	0.05
Breast cancer	−0.87	0.37	0.02
Treatment with a DOAC	−0.42	0.21	0.04
DVT only as index event	0.54	0.14	<0.01

Abbreviations: DOAC, direct oral anticoagulants; DVT, deep vein thrombosis.
[a]Restricted cubic splines were used with 3 knots located at age 49, 65, and 78.

 © 2024. The Author(s).

Metastatic disease yes or no

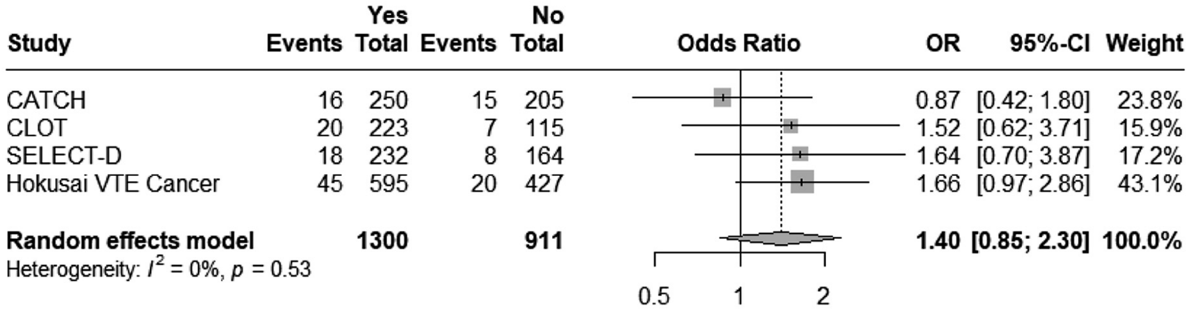

Use of antiplatelets yes or no

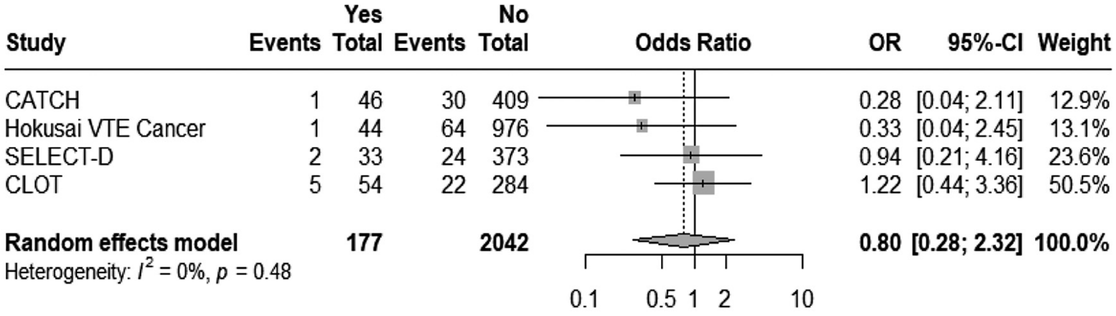

Index event deep vein thrombosis only

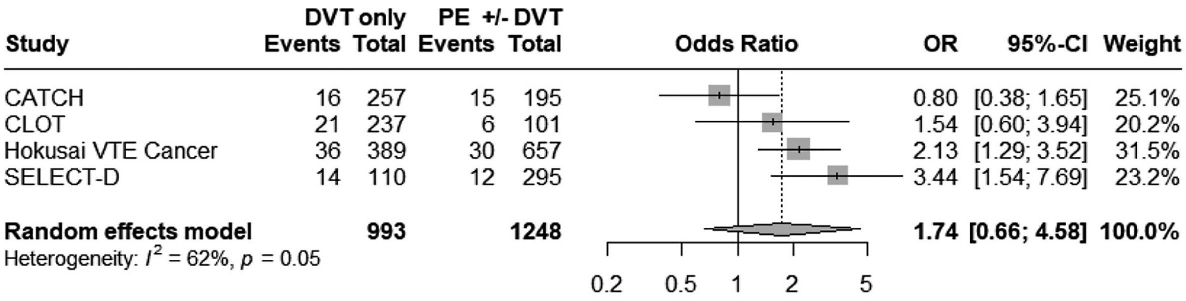

Sex

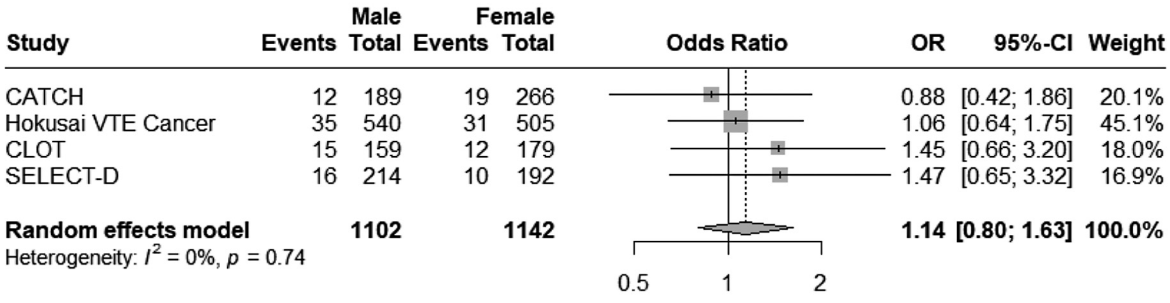

Supplementary Fig. S1 Forest plots of relevant binary candidate predictors.

 © 2024. The Author(s).

Platelet count >350 × 10^9/L

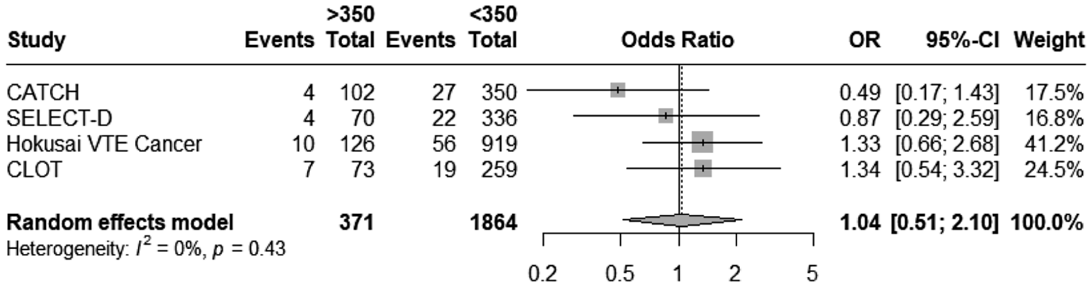

Eastern Cooperative Oncology Group score 0 vs. 1–2

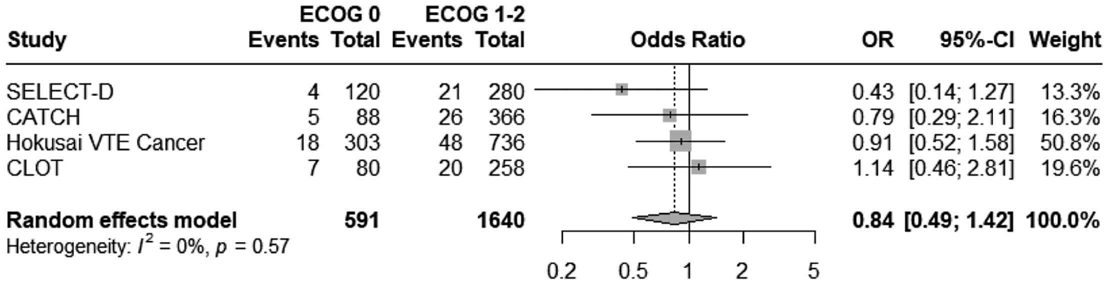

Hematological cancer vs. solid cancer

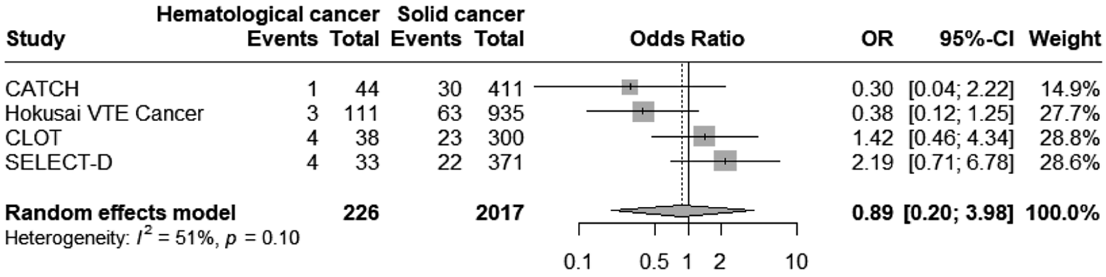

Li cancer classification high and very high risk cancer types vs. intermediate- and low-risk cancer types

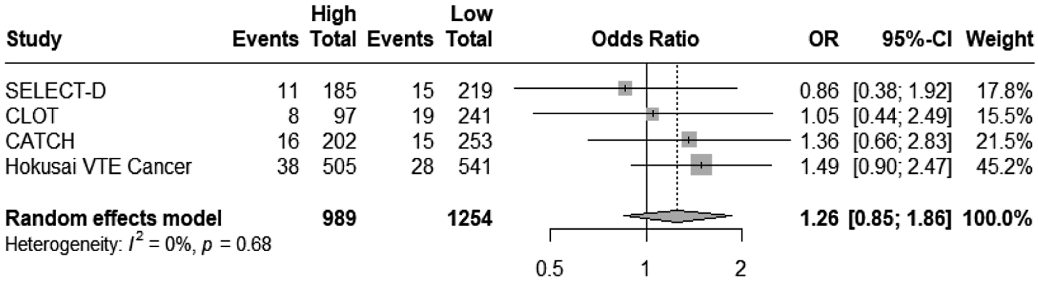

Supplementary Fig. S1 (*Continued*)

 © 2024. The Author(s).

Breast cancer

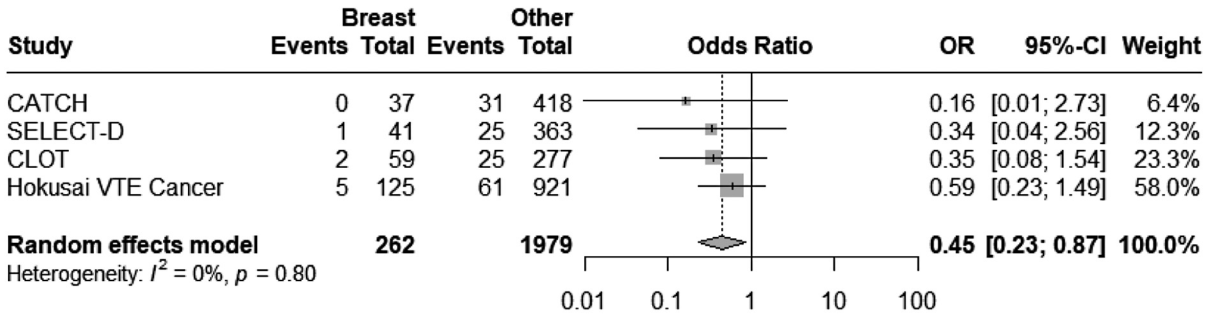

Lung cancer

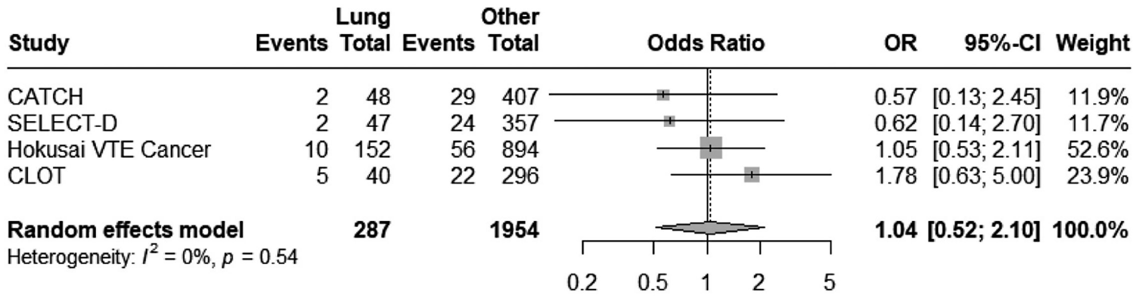

Hepatobiliary cancer

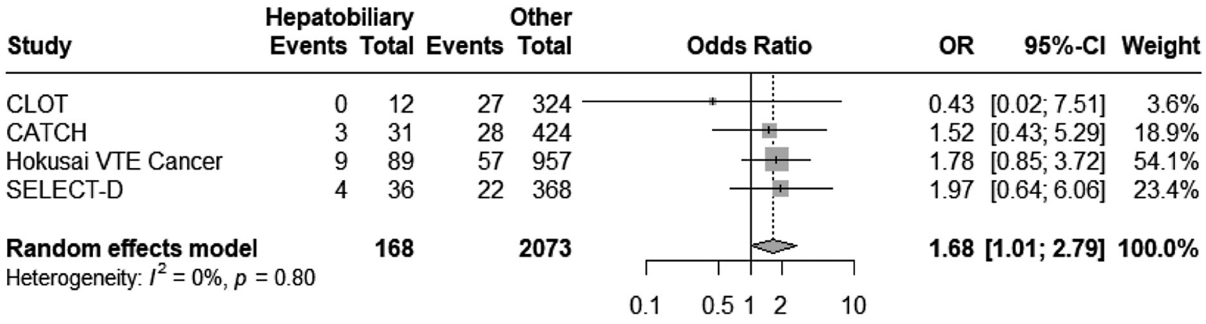

Pancreatic cancer

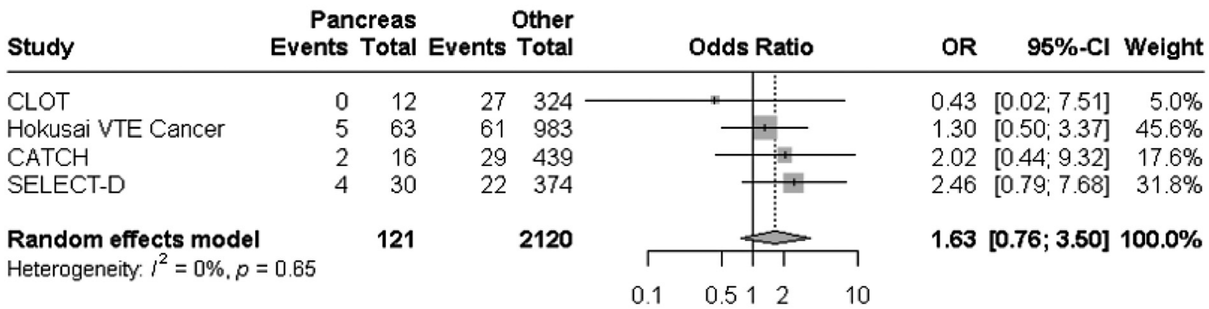

Supplementary Fig. S1 (*Continued*)

 © 2024. The Author(s).

Upper gastrointestinal cancer

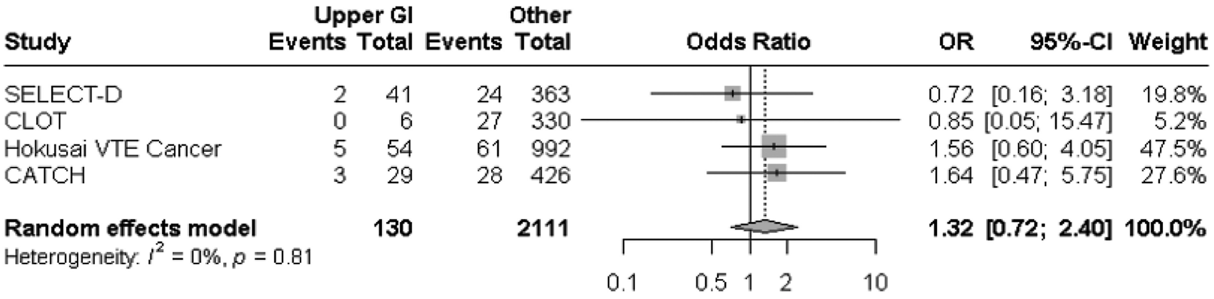

Genitourinary cancer excluding prostate cancer

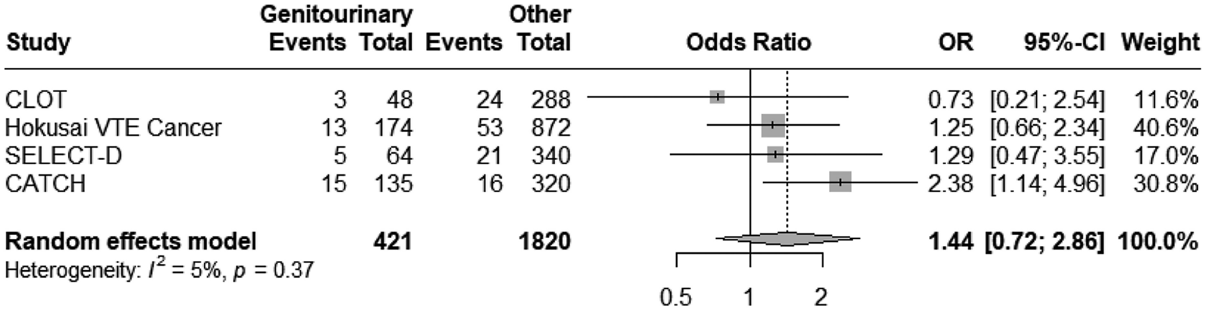

Gynecological cancer

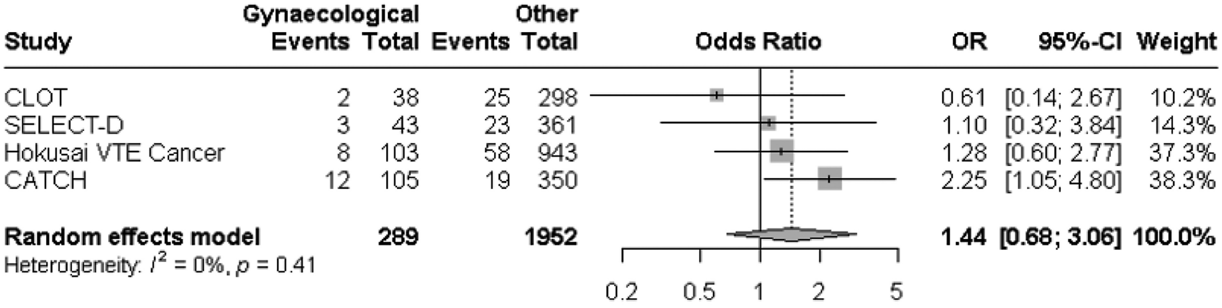

Use of LMWH (CLOT and CATCH vs. VKA; Hokusai and Select-D vs. DOAC)

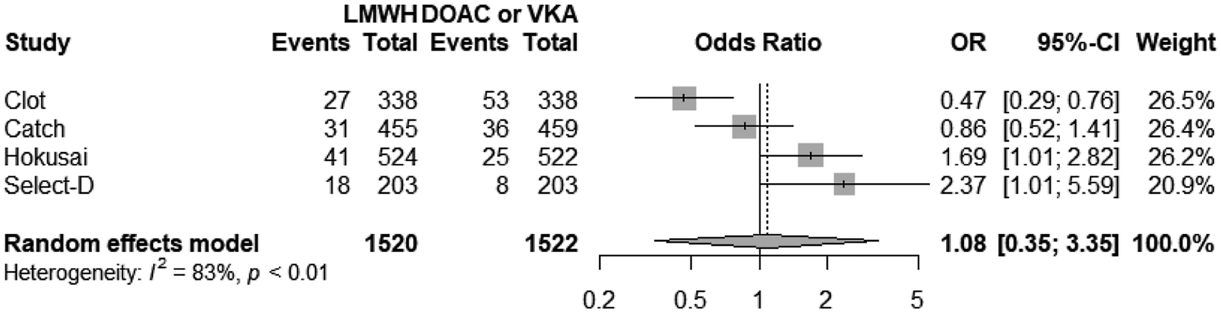

Supplementary Fig. S1 (*Continued*)

 © 2024. The Author(s).

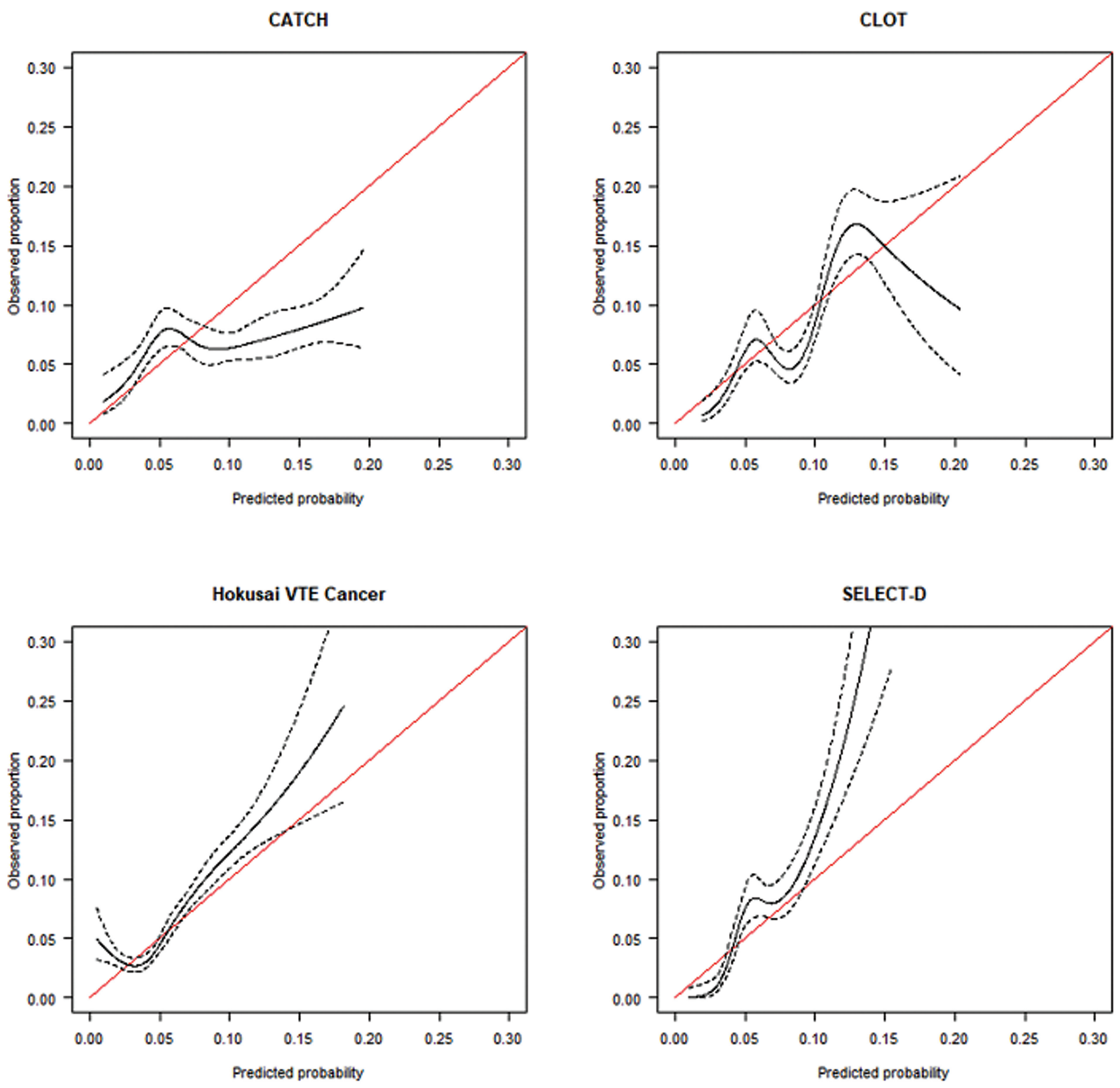

Supplementary Fig. S2 Calibration of the model in each study

 © 2024. The Author(s).
